# Fracture-matrix fluid exchange in oil-bearing unconventional mudstones

**DOI:** 10.1038/s41598-023-48688-z

**Published:** 2023-12-07

**Authors:** Johnathan Moore, Dustin Crandall, Sean Sanguinito, John J. Valenza

**Affiliations:** 1 U.S. Department of Energy, National Energy Technology Laboratory, Morgantown, WV 26507 USA; 2 Research Division, ExxonMobil Technology and Engineering Co., Annandale, NJ 08801 USA

**Keywords:** Carbon capture and storage, Condensed-matter physics, Soft materials, Applied physics, Climate sciences, Materials science, Chemical engineering, Mechanical engineering

## Abstract

The poromechanical properties of unconventional reservoir materials are in large part dictated by their mineralogy. Since these properties govern the response to stress experienced during hydraulic fracturing, fluid production, and fluid injection, they play a central role in the formation of microcracks or bedding delaminations which ultimately dominate mass transport. In this work we study access to the porosity of end member unconventional reservoir materials, where the end members are predominantly dictated by carbonate content. Access to the porosity is quantified using state of the art 3D x-ray computed tomography coupled with physics informed data analytics. Xenon gas, which attenuates x-rays, provides a spatiotemporal map of access to the porosity. The accessible porosity is quantified over a range of net confining stress relevant to the manmade disturbances listed above. These experiments demonstrate that heavily carbonated mudstones are nearly impermeable at the core (~ cm) scale, while carbonate free analogues afford better access to the microstructure. Consistent with previous qualitative 2D radiographs, access to the interior of the clastic mudstones is first observed along planar microcracks, followed by slow penetration into the surrounding matrix. Physics informed data analytics of the 3D tomography measurements presented here show that these microcracks do not permit uniform access to the adjacent rock matrix. In addition, variation of the effective pressure elucidates the mechanisms that govern fracture/matrix fluid exchange. Under conditions consistent with hydrocarbon production fluid accumulates in the immediate vicinity of the nearest microcrack. While there is clear evidence that, as intended, part of this accumulation is from the more distant matrix, fluid is also squeezed out of the microcrack. The fluid build-up at the microcrack indicates that migration out of the rock is hindered by the coupled poroelastic response of the microcrack and adjacent rock matrix. We show that these mechanisms ultimately account for the meager oil recovery factors realized in practice. These insights have implications for making reservoir scale predictions based on core scale observations, and provide a basis for devising new asset development techniques to access more porosity, and enhance fluid extraction. Finally, these findings shed light on key features and mechanisms that govern shale storage capacity, with relevance to other important industrial processes, such as geologic CO_2_ storage.

## Introduction

Unconventional hydrocarbon resources are experiencing a renaissance due to the ability to extract liquids at economically viable rates. In fact, recent research demonstrates that these assets are competitive with other world class (including deepwater) assets when compared on the coupled basis of development cost and greenhouse gas emissions associated with fluid production^1,2^. In particular, the carbon footprint of shale oil production in the Permian Basin is on the order of 20 kg CO_2_/barrel of oil^3^ which is lower than that of other unconventional (eg. Bakken—40 kg CO_2_/barrel^4^) or conventional hydrocarbon assets (e.g. Alaska, North Slope—30 kg CO_2_/barrel^5^). As a result, there is a very high probability that these assets will be fully developed^2^. This is the case even though these resources exhibit substantially lower recovery factors (3–10×) than that from conventional hydrocarbon reservoirs. We utilize state-of-the-art x-ray computed tomography imaging and machine learning to elucidate the mechanisms that trap (> 90%) fluid in the rock after exposure to conditions meant to mimic unconventional resource development. This insight may be utilized to devise techniques to increase fluid recovery and ultimately reduce the associated carbon footprint.

While previous work on gas shale focused on access to^6^, or transport through^6,7^, the porosity, and field scale production kinetics^8^, there is less information on the mechanisms that govern fluid extraction from the lower thermal maturity oil bearing resources. This work focuses on how these reservoir materials behave when exposed to stress states experienced during hydraulic fracturing and fluid production. In addition to providing a basis for deriving techniques to increase fluid production, our results suggest care must be taken when utilizing data from core analysis (e.g. like pressure dependent permeability^9–12^) in reservoir scale predictions. Finally, since unconventional oil wells rapidly (~ 18–24 mos.) reach the end of primary production, they represent targets for enhanced oil recovery (EOR), and geologic CO_2_ sequestration. This work also provides insight on the potential value of EOR, and mechanisms that may be exploited to enhance CO_2_ trapping.

## Materials and methods

### Rock samples

The unconventional reservoir materials used in this study are from the Delaware basin, which is a subset of the Permian Basin predominantly located in western Texas and southeast New Mexico, USA. The Permian basin hydrocarbon system formed in a deep inland water way that was surrounded by a carbonate reef^13^. As sediment filled the basin to form the Wolfcamp interval, episodic turbidite driven shedding of carbonate material from the marginal reef produced ~ 7000 ft of carbonate interbedded mudstone. The overlying tight sands exhibit similar interbedding. In both cases the rocks have a relatively narrow compositional envelope (see supplementary information, Fig. S-1)^14–18^. Therefore, it is possible to identify end members to assess the effect of composition on rock behavior. The rocks studied here differed primarily in the amount of carbonate (Fig. S-1), varying from high argillaceous:low carbonate (HA:LC) to low argillaceous:high carbonate (LA:HC). In addition, we utilized a rock with intermediate composition labeled moderate argillaceous:moderate carbonate (MA:MC).

### Material characterization

Portions of each sample were removed and utilized in various complimentary characterization techniques, all of which are described in the supplemental information. In particular, the composition of the rocks was inferred from x-ray diffraction (XRD), electronprobe microanalysis (EPMA), and elemental dispersive spectroscopy with a scanning electron microscope (SEM). The latter two techniques also indicate the rock morphology at the cm and 0.01 cm length scale, respectively. The pore size distribution was inferred from mercury intrusion capillary porosimetry (MICP).

### X-ray computed tomography (XCT)

The cylindrical plugs, 3.81 cm diameter by ~ 3.81 cm long (Table 1), are taken from whole core (diameter ~ 15.2 cm) that is extracted at depth, then raised to the earth’s surface. The plugs are oriented with the symmetry axis parallel to the bedding direction, and all fluid flow is due to pressure gradients imposed parallel to the symmetry axis (e.g. along bedding). The plugs were loaded into Buna-N sleeves and inserted into a biaxial carbon fiber Hassler style core holder. The Buna-N sleeve isolates the sample from the water used to impose a hydrostatic confining pressure (*P*_conf_), while also permitting independent control of the pore pressure (*P*_pore_) using either Nitrogen (N_2_, minimum purity 99.5%) or Xenon (Xe) gas. All pressures (*P*_conf_, *P*_pore_) were controlled with Teledyne Isco 100DX syringe pumps. The experiments were performed at ambient temperature (20 < T °C < 22) and the effective pressure, *P*_eff_ = *P*_conf_-*P*_pore_, was varied with N_2_ to test for mechanical alteration to the mudstones, and Xe to study access to the porosity. Pressures, flow rates, and temperatures were recorded using standard data acquisition techniques with National Instruments Labview™ at a rate of 0.2 Hz.

**Table 1:** Relevant sample dimensions and properties. Porosity and bulk density from mercury intrusion results reported in the supplementary information, and an order of magnitude estimate for the pore size that controls mass transport, 2*r*_P_.

Sample ID	Diameter (cm)	Length (cm)	Porosity (%)	Bulk Density (g/cc)	2*r*_P_ (nm)
HA:LC	3.81	2.84	9.93	2.41	10^1^
MA:MC	3.81	3.81	2.10	2.64	10^1^
LA:HC	3.81	3.45	1.20	2.72	10^0^

A NorthStar Imaging Inc. (NSI) M5000 Industrial computed tomography (CT) scanner was used to capture spatiotemporal evolution of the x-ray absorption. Acquisition was performed at a voltage of 185 kV and current of 400 µA using a polychromatic source generated by a tungsten target. Each scan consisted of 1,440 projections, with each projection representing the average of 12 measurements taken over 2 s acquisition time. Reconstruction of the volume was performed using NSI’s EFX-CT software. A consistent grayscale value was used for all x-ray CT reconstructions to enable comparison of results from sequential scans. Reconstructed volumes were exported as a series of 16-bit tiff images with a voxel resolution of (23.6 µm)^3^. Each CT scan took approximately 2 h and 35 min. A schematic of the setup to concomitantly manipulate *P*_eff_ and perform CT scans is shown in the supplemental information Fig. S-2.

**Figure 1:**
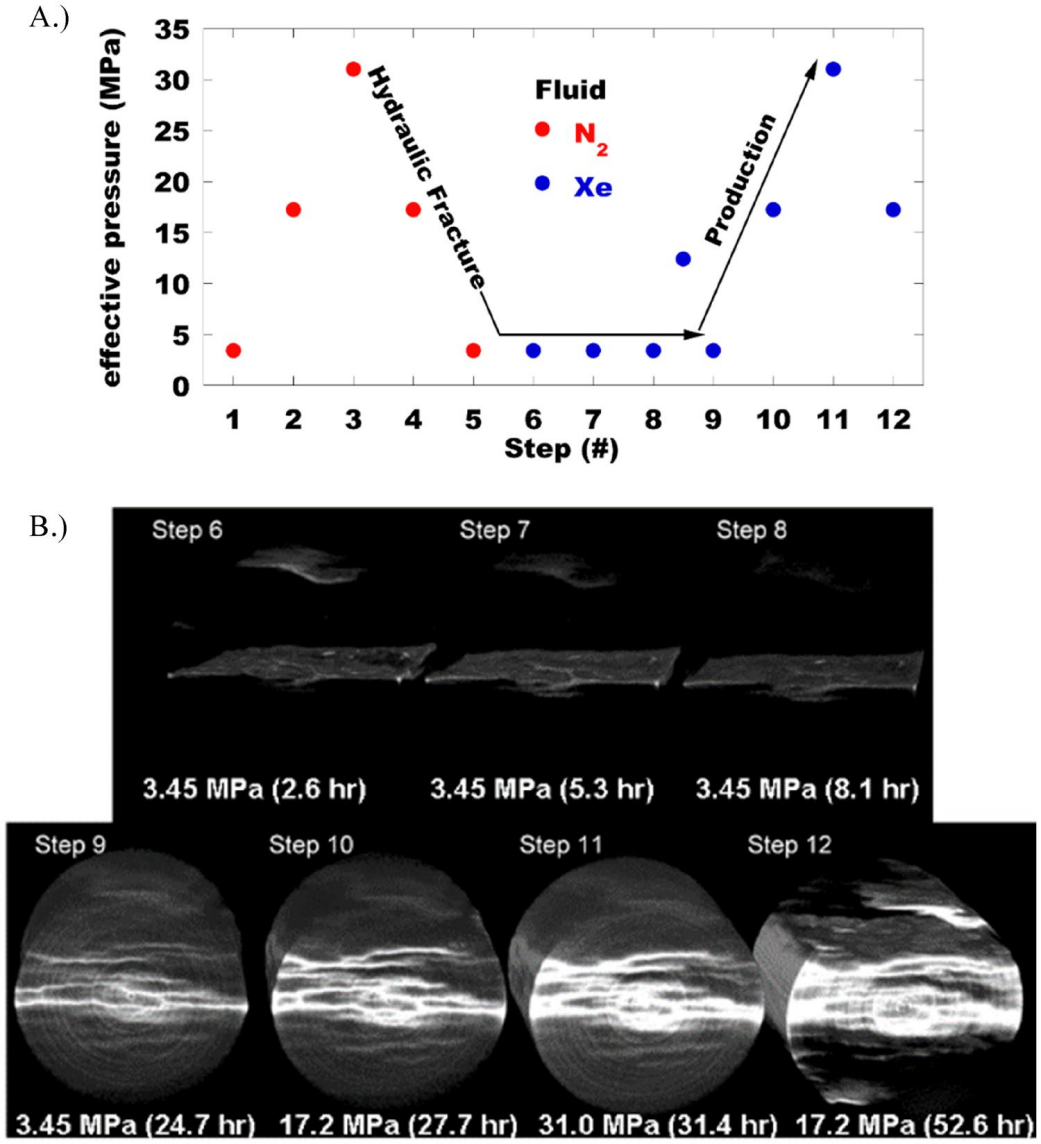
Experimental protocol followed with each sample. The sample was exposed to a series of effective pressure cycles (**A**), first with Nitrogen (N_2_), then with Xenon (Xe). After the effective pressure stabilized a tomography measurement was performed (**B**). The computed tomography images collected with N_2_ (not shown) were analyzed to check for structural changes caused by varying the effective stress. As indicated in panel (**A**), a small leak in the upstream pore fluid pump resulted in a relatively small effective pressure increase between steps 8 and 9. The reconstructed core volumes shown in panel (**B**) consist of the difference between the x-ray CT measurements with N_2_ (step 5) and Xe for the various effective stress conditions imposed through steps 6–12. The difference images, where white (black) indicates maximum increase (decrease) in x-ray absorption, map out the spatial distribution of Xe as a function of effective pressure. The time indicated in each panel is the cumulative experimental time at which the CT scan was finished for each step.

Samples were subjected to a series of discrete pore pressures using N_2_ (Fig. 1A, steps 0–5). After switching the pore fluid to Xe, *P*_eff_ was held constant for 24 h followed by a similar pressure cycle (Fig. 1A, steps 6–12) where the increase in *P*_eff_ is meant to mimic conditions consistent with fluid production. In the case of the final step *P*_eff_ (step 11 → 12) was reduced by lowering *P*_conf_, whereas previous manipulation of *P*_eff_ was accomplished by varying *P*_pore_. The highest pore pressure, *P*_pore_ = 31 MPa, is above the critical point for both gases at ambient temperature. The confining pressure, *P*_conf_, was held constant at 34.5 MPa except for steps 1, 2, and 12 where the hydrostatic pressure was 6.9, 20.7, and 20.7 MPa, respectively. As discussed below, we substituted Xe for N_2_ in the second *P*_eff_ cycle to exploit the increase in x-ray attenuation and improve the sensitivity to the spatial distribution of fluid saturation in the core. A CT scan was taken at each pressure step. The N_2_ pressure cycle was used to evaluate both the mechanical changes in the cores and the degree of change to the transmissivity across the core as a function of *P*_eff_. The difference between CT scans taken at each effective stress with Xe (Fig. 1B, steps 6–12), and the final scan measured with N_2_ (step 5), were interrogated to determine how *P*_eff_ impacts fluid migration.

### Finite element calculation

Comsol Multiphysics software was utilized to model the 2D poromechanical response under conditions that mimic fluid production. The constitutive models are linear elasticity and Darcy’s law to account for the mechanical response and flow through porous media, respectively. These material responses are coupled through the poroelasticity multiphysics module. Details about the model including, the governing equations, definition of the material properties, the relevant geometries and boundary conditions are outlined in the supplementary information.

## Theory and analysis

### Xenon flooding

X-rays have broad utility for non-destructive testing. For the core experiments x-rays provide the unique advantage of being sensitive to the evolution of pore fluid composition as the effective stress is varied. This sensitivity is due to x-ray absorption, which is governed by the photoelectric effect, as described by the Beer–Lambert law^19^:

1 $$\frac{I(x)}{{I(0)}} = e^{ - \mu x}$$

where, *I*_0_ = *I*(0) is the intensity of the x-rays entering the sample, *I*(*x*) is the intensity at any point in the sample having traveled a distance, *x*, through the sample, and *μ* is the spatial attenuation coefficient. Attenuation due to the photoelectric effect is strongly dependent on the atomic number, *Z*^19^,

2 $$\mu \sim\frac{{\rho^{2} Z^{4} }}{{mE^{3} }}$$

where *ρ* is density, *m* is atomic mass, and *E* is the x-ray energy. Equation 2 demonstrates that using a fluid with a relatively large Z is the best way to track spatiotemporal pore fluid composition. These so-called contrast agents are widely used in CT, both medically^20^ and experimentally^21,22^. In petrophysics, Xe is particularly useful because it is a gas at low pressure, has a relatively high atomic number (Z = 54), and is generally inert. The higher gas mobility and attenuation impart the ability to discern relatively tight flow pathways and areas of a rock that would not be accessible to traditional liquid contrast agents, such as potassium iodide dissolved in water.

### Image processing and analysis

A full core CT scan consumes 12 GB of memory. So the data sets were scaled by 50% in all 3 dimensions to make the file size more manageable during post-processing (see supplementary information—Fig. S-3), which doubled the voxel resolution to (47.2 µm)^3^. Noise associated with the polychromatic x-rays was reduced with a 3D Gaussian blur using the image processing program Fiji^23^. To emphasize the location of Xe in the sample we utilized the following image analysis workflow (see supplementary information—Fig. S-3). First a baseline position specific CT_N2_(x,y,z) number was measured with N_2_ in the pore space (e.g. Fig. 1—step 5). Next, the sample was exposed to Xe and the associated CT_Xe_(x,y,z) number corresponding to Xe infiltrated rock was accentuated by taking the difference with CT_N2_ using the image calculator utility in Fiji. Prior to taking the difference, the images were registered and converted to 32-bit so increases (decreases) in CT number indicate an increase (decrease) in local Xe concentration.

The difference images (Figure 1B) clearly show the change in attenuation associated with variation in Xe concentration throughout the sample. However, the difference images exhibited a relatively continuous evolution in Xe concentration in volumes accessible to the gas. Therefore, simple thresholding will not identify all the pixels that exhibited a change in Xe concentration. As a result, we used *ilastik*^24^, a python-based neural network characterization program for image parsing, to classify all the pixels in our difference images. This pixel-based characterization utilizes user provided training data combined with filters and a random forest classifier to assign every pixel to a user defined class. The training process was iterative, permitting the user to add training data and thus improve the classifier accuracy. With this approach we identified three segmentation classes: the rock matrix, fractures, and the rubber sleeve/air (see supplementary information—Fig. S-4). The classifier was manually trained using the drawing tools in *ilastik*, dynamic classification monitoring was performed using the live update feature to reduce low opacity zones across the image volumes, and the segmentations were refined to reduce uncertainty. The recursive nature of adding class annotations, or training markers, allowed the random forest classifier to extract the three classes of interest accurately and consistently.

**Figure 2:**
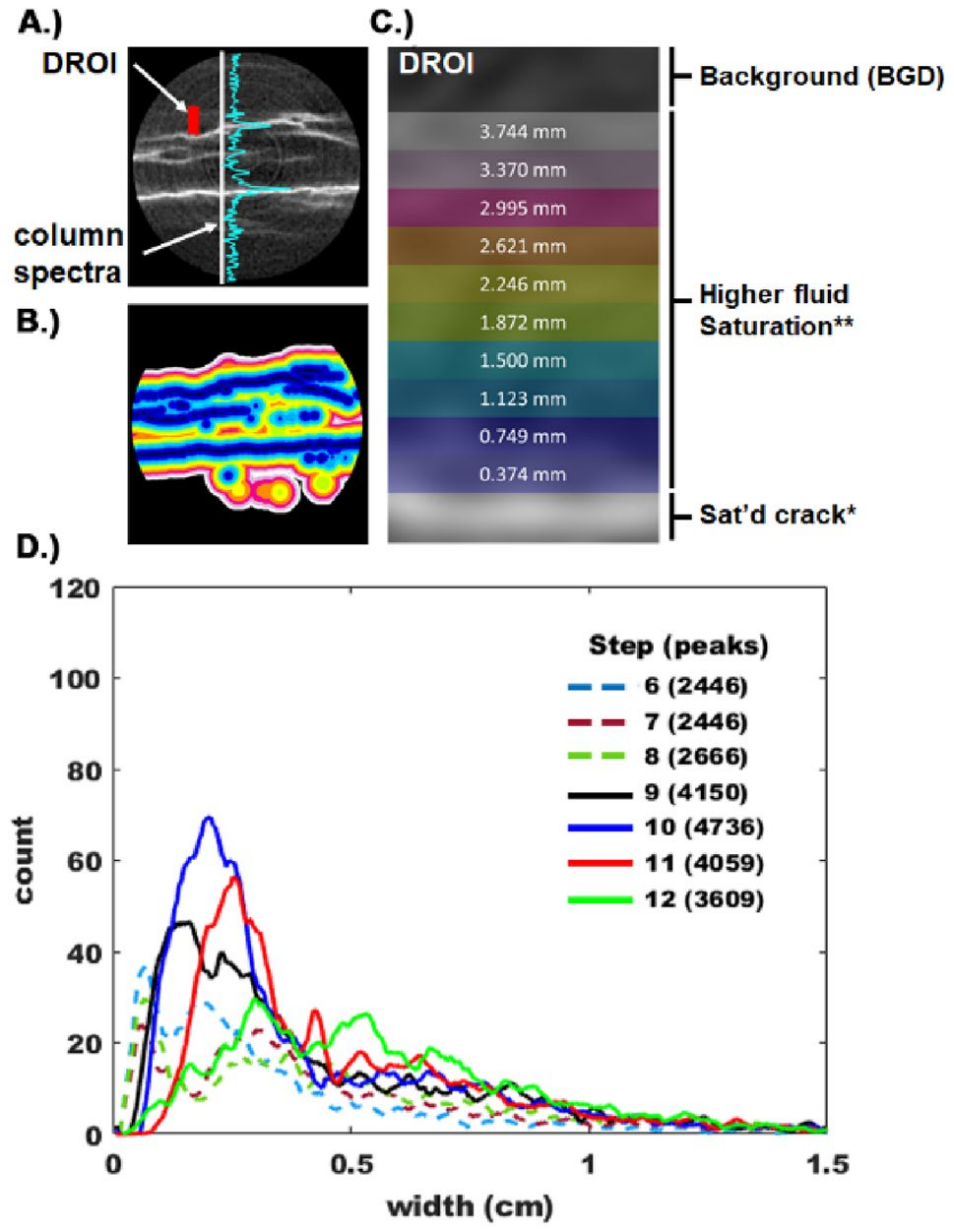
(**A**) Cylindrical cross section of difference image indicating the concentration of Xe in delamination cracks, and the enhanced fluid saturation in the vicinity of the delaminations. By analyzing the column spectra we determine that Xe concentration is enhanced over a maximum length scale of ~ 7.5 mm from each crack (see **D**) below). This length scale is utilized to identify the diffusive region of interest (DROI) throughout the core. (**B**) The DROI is determined for each crack pixel using the morphLibJ Fiji plugin. (**C**) Schematic indication of half of a single DROI, and length scales used to quantify the Xe concentration in the vicinity of the crack. (**D**) Histogram of peak widths determined from column spectra shown in (**A**). While most cracks are characterized by an elevated Xe concentration spanning 0.2 cm, the Xe concentration is elevated over a maximum length scale of 0.75 cm. The step number corresponds to the equilibrium pressures shown in Fig. 1A, and the number in parenthesis indicates the total number of peaks identified by the peak fitting routine.

The greatest extent of Xe penetration into the sample were concentrated around planar features, hereafter referred to as delaminations, characterized by length scales on the same order as the core diameter (Fig. 2A). The extent of the Xe infiltrated diffusive region of interest (Fig. 2A—DROI) was determined from the difference images by analyzing the intensity spectra corresponding to a column of pixel values (Fig. 2B, e.g., perpendicular to the delaminations). We utilized a peak fitting algorithm to identify, then characterize, the peaks with a modified Lorentzian^25^:

3 $$I(y) = \frac{\Gamma }{{(y - y_{0} )^{2} - \Psi }}$$

where y_0_ is the peak (pixel) location along the spectrum, Γ, and Ψ are fitting parameters to account for the peak height, and width. Once the fit was optimized, the width was determined from equation 3 by setting *I*(*y*) equal to one half of a standard deviation of the spectrum background noise^26^. We also utilized equation 3 to remove the peak from the spectrum. This eliminated the possibility of double counting peaks and facilitated rapid identification of neighboring and overlapping peaks. We applied this approach to 11 spectra that were spaced 50 pixels apart on the average of 4 successive slices (e.g. total of 158 slices analyzed or 1738 spectra) along the symmetry axis of the reconstructed core volume. The area interrogated in this manner is centered on the cylindrical cross section (e.g. the central column of pixel intensities and 5 columns on either side). The histogram of peak widths inferred from this recursive exercise is shown in Fig. 2D for steps 6–12 (Fig. 1A). These results demonstrate that the average peak width varies with *P*_eff_, and the widest DROI is approximately 0.75 cm.

With the extracted fracture/bedding plane features identified using the *ilastik* characterization scheme, we used the largest DROI width to identify all pixels within the DROI throughout the core. This is accomplished with the Morphological Filters 3D Dilation process from the MorphoLibJ^27^ imageJ plugin. This routine dilates the isolated delaminations as shown in Fig. 2B. The DROI were subdivided in to 10 equidistant regions from the delamination up to a half-width of 0.375 cm (Fig. 2C).

Identifying each pixel associated with the DROI permitted analysis of the CT intensity as a function of proximity to the delamination throughout the core volume as a function of *P*_eff_. In general, the grayscale values did not exceed 1500 (e.g. no more than 0.4% > 1500). Therefore, the histograms for each effective pressure were split into 15 equal bins each representing a range of 100 greyscale values. The 10 subdomains of the DROI were then interrogated by thresholding all 15 bins out of each image set. The proportion of the DROI volume corresponding to each grayscale range was determined by sequential thresholding using the Fiji plugin Bonej^28^.

## Results

### Structure

**Figure 3:**
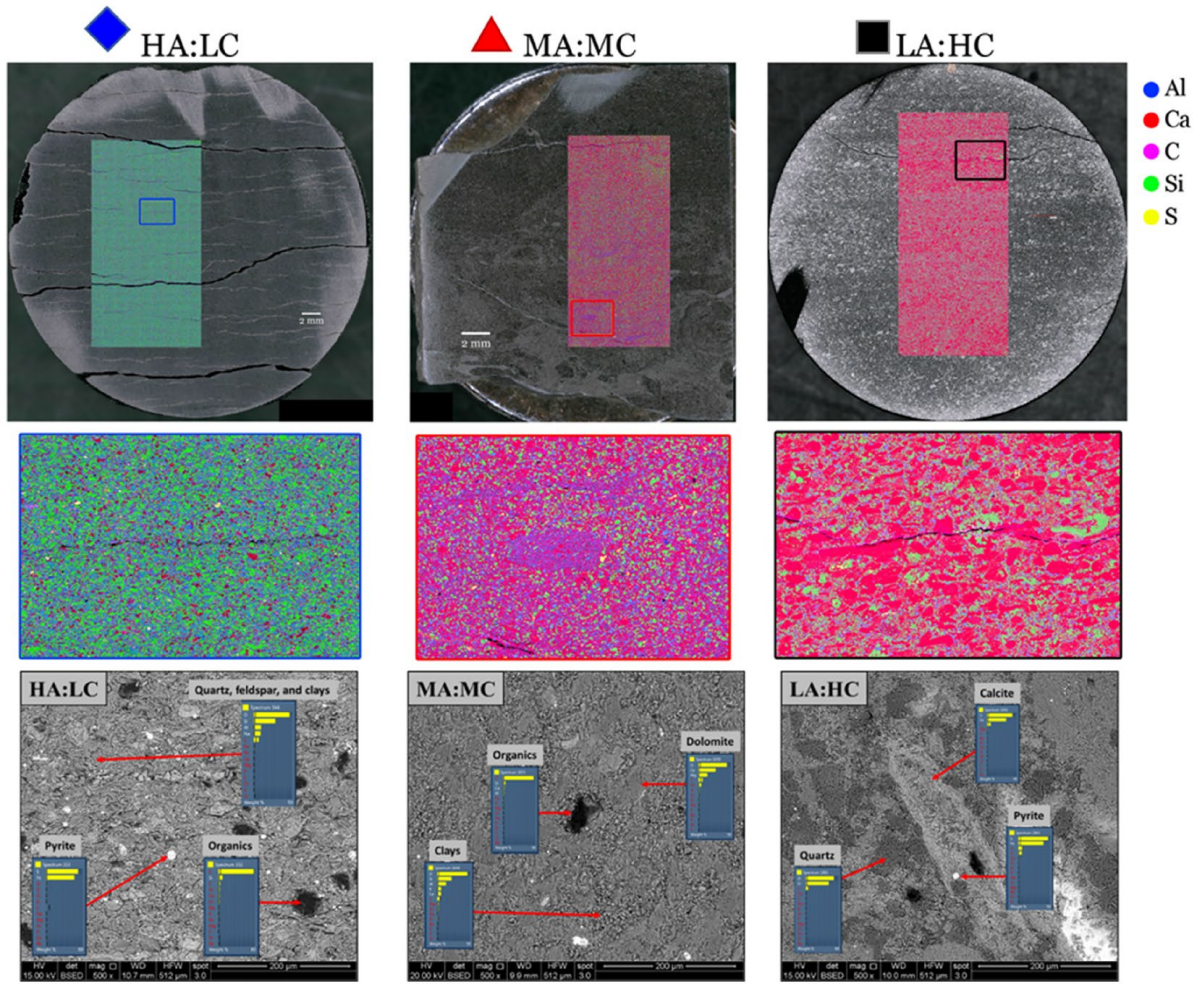
*Top row*: optical images (diameter ~ 3.81 cm) with elemental map (~ 1 cm × 2.5 cm) overlay showing that the primary compositional variation is an evolution of the silt/carbonate ratio, where the legend on the right indicates color scheme used in the elemental map. *Middle row*: Magnified view of highlighted region on elemental maps showing fine resolution (~ 5 μm) of EPMA technique and variation in breadth of grain size. *Bottom row*: SEM micrographs with energy dispersive x-ray spectroscopy (EDS) at various points to verify changes in composition and grain size.

The EPMA and SEM results are shown in Fig. 3. The elemental maps show that our expectations from the XRD results (Fig. S-1, Supplementary information) are confirmed. Sample HA:LC is predominantly made of silt (Si for SiO_2_), clay (Al) and organic carbon (C for solid or immobile HC). The sample also contains a very small, uniformly distributed quantity of carbonate (Ca for CaCO_3_) and pyrite (S for FeS_2_). The other two samples exhibited drastically different elemental maps. Sample MA:MC exhibits an equally fine microstructure with more prevalent and slightly larger carbonate inclusions (solid red circular features), while sample LA:HC contains even larger, and more prevalent carbonate inclusions. Both samples are also characterized by a uniform carbonate coating throughout the microstructure. In contrast, the HA:LC sample is a classic siliceous mudstone with small, uniformly dispersed, carbonate features that are likely carbonate cement formed during diagenesis.

The SEM images corroborated our scheme of using the elements identified with the electron microprobe as mineral proxies; the images show that the microstructure of HA:LC and MA:MC exhibited similar fine grain texture and the rocks contain silica, clay, organic carbon, carbonate, and pyrite. As described above, LA:HC consists of larger carbonate inclusions, and the silicate phase is a silicate cement that forms during diagenesis.

The mercury intrusion results are shown in the supplementary information (Fig. S-5A), along with data corresponding to a conventional Berea sandstone. The porosity in Table 1 is estimated by multiplying the cumulative intrusion volume by the bulk density. We note that the two carbonated samples have very low porosity, and that these values are significantly lower than that (~ 0.10) for three-dimensional percolation^29^. This is the case even though mercury intrusion was performed on (~ 1–2 mm) granulated sample. So, this porosity may be significantly higher than that measured on the whole plug. Moreover, unconventional reservoir materials have significantly lower porosity than a conventional sandstone. In addition, the characteristic pore size that governs mass transport through the porosity, *r*_P_, may be estimated from the inflection in the intrusion volume^30^ (Table 1). Unconventional reservoir materials contain significantly smaller pore sizes than the sandstone. Finally, considering the unconventional rocks alone, these results demonstrate that carbonation results in a further reduction in the porosity, and the pore size distribution (Table 1).

### Transport

The N_2_ pressure cycles (Fig. 1A) were completed on all three samples listed in Table 1. The difference images for the CT measurements corresponding to the end of each step in the N_2_ pressure cycle had no notable features, consisting of an identical flat background with similar statistics. This indicates that the variation in CT number throughout the rock due to mechanical response was below the detection limit of our measurement. At the end of the N_2_ cycle, when the effective pressure was at a minimum (500 psi) the pore fluid was switched to supercritical Xe (99.99% purity). The argillaceous mudstone (HA:LC) was the only sample that exhibited the ability to transmit N_2_ or Xe through the core (see supplementary information, Fig. S-5B). Due to the low, likely disconnected, porosity and nm-scale pore size, the carbonated cores (MA:MC and LA:HC) were impermeable over the timeframe used for injection. In addition, the x-ray CT images for MA:MC and LA:HC corroborated there was no fluid substitution as determined from the difference images. The higher transmissibility in HA:LC was due to the presence of multiple interconnected delaminations and a single fissile fracture in the center of the sample that spanned the core length. After exposing HA:LC to Xe, three CT scans were taken over the following 8 h (Fig. 1B, Step 6–8). Since *P*_eff_ was held constant while switching the pore fluid to Xe, the consistency in these difference images indicates that little to no diffusive exchange occurs during a single CT scan (2 h. 35 min.). Between steps 8 and 9, approximately 16 h after the initial high pressure Xe exposure, there was a leak in the injection pump. As a result, the upstream pore pressure slowly dropped to *P*_Pore_ ~ 22.1 MPa. Then the core was isolated from the leaking pump and additional Xe was injected to increase *P*_pore_ to 31.0 MPa before the CT scan at step 9. Next, *P*_eff_ was increased, by decreasing *P*_pore_ on the downstream side of the sample during step 10 and 11. Finally, *P*_eff_ was reduced by reducing *P*_conf_ (step 12), which is a manipulation of the stress state that is not possible in practice. It is important to note that the equilibration times prior to starting the CT scan but after manipulating *P*_eff_ for steps 10 and 11 (30–60 min.) and that for step 12 (19 h).

## Discussion and conclusions

Over the core scale rock composition dictates access to the porosity. The MICP results (Fig. S-5A) indicate that the carbonated rocks are characterized by reduced pore size, and lower porosity. Carbonation reduces pore size and porosity as the tight microcrystalline rock fabric is fortified by secondary carbonate mineralization during diagenesis. This tight, likely discontinuous, microstructure does not permit measurable mass transport over the length (cm) and time scale of our experiments (Fig. S-5B). Conversely, the siliclastic mudstones are significantly more transmissive, especially near fracture. Altogether this suggests that most fluid production is from the clastic fraction of the reservoir (Supplementary information—Fig. S-1).

**Figure 4:**
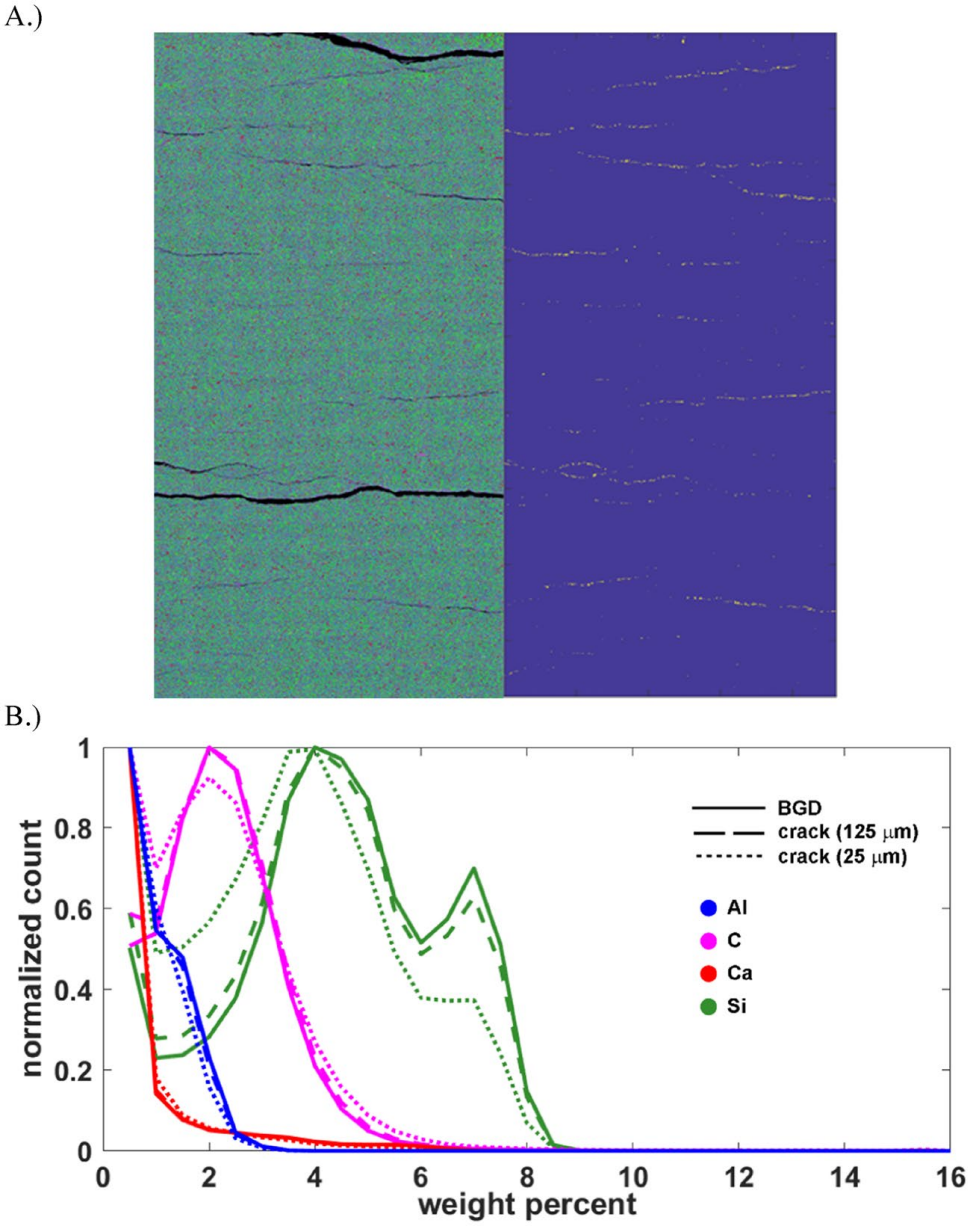
(**A**) *Left*: Elemental map for sample HA:LC. *Right*: crack pixels only, excluding the large cracks that formed when the end-trim was removed. Crack pixels were identified by simple thresholding. (**B**) Distribution of representative elements for the entire sample and in the vicinity of the crack pixels for two different length scales. Here we assume each element is a (proxy) in the following manner: Al (clay), C (organic C), Ca (inorganic C) and Si (silt and sand).

Access to the porosity in the HA:LC mudstone is through bedding parallel delaminations, features that are not present on the carbonated analogues (Fig. 3). The gas leak between steps 8 and 9 mimicked a huff-and-puff cycle, encouraging the pore fluids (N_2_ and Xe) to coinhabit the porosity throughout the core through convective mixing. The difference image corresponding to step 9 (Fig. 1B) highlights several bedding-parallel delaminations corresponding to an increase in Xe concentration, and the core volume appears mottled indicating that Xe does not uniformly access the porosity. Therefore, Xe cannot access the entire core even over a length scale corresponding to the separation between delaminations (< 0.5 cm). The delaminations tend to form in thin planes that are both slightly enriched with organic carbon, and deficient in silica. This is demonstrated in Fig. 4 where we interrogate the elemental map shown in Fig. 3 to determine the elemental composition proximal to the delaminations.

At the end of step 9 the delaminations and proximal rock matrix were accessible under conditions mimicking hydraulic fracturing operations corresponding to the lowest *P*_eff_. As the pore pressure at the face of the core is subsequently reduced in steps 10 and 11, mimicking hydrocarbon production, the delaminations are further accentuated and new secondary delaminations appear (Fig. 2D—average peak width and peak count increase). At the core scale the rock matrix retained a mottled appearance throughout the core. Finally, when the confining stress was reduced during step 12 the primary delaminations are even further highlighted, the secondary delaminations fade, and Xe appears to be completely removed from portions of the core. This latter stress manipulation is akin to relieving the stress due to the sediment overburden. This is not indicative of any operation that may be employed in practice.

**Figure 5:**
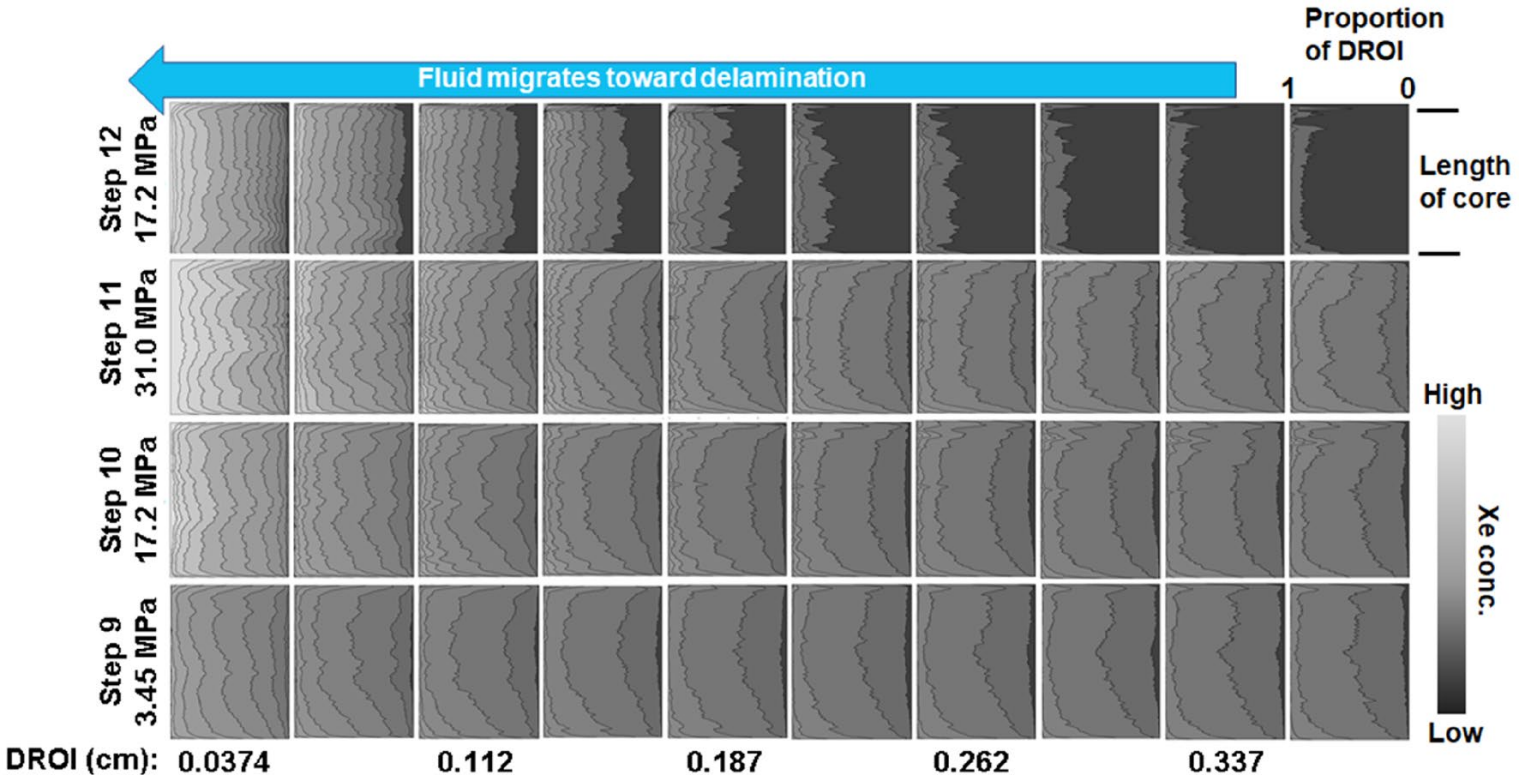
Xenon concentration profile as a function of position in the diffusive region of interest (DROI) adjacent to the delaminations present throughout the sample as a function of the indicated effective stress.

**Figure 6:**
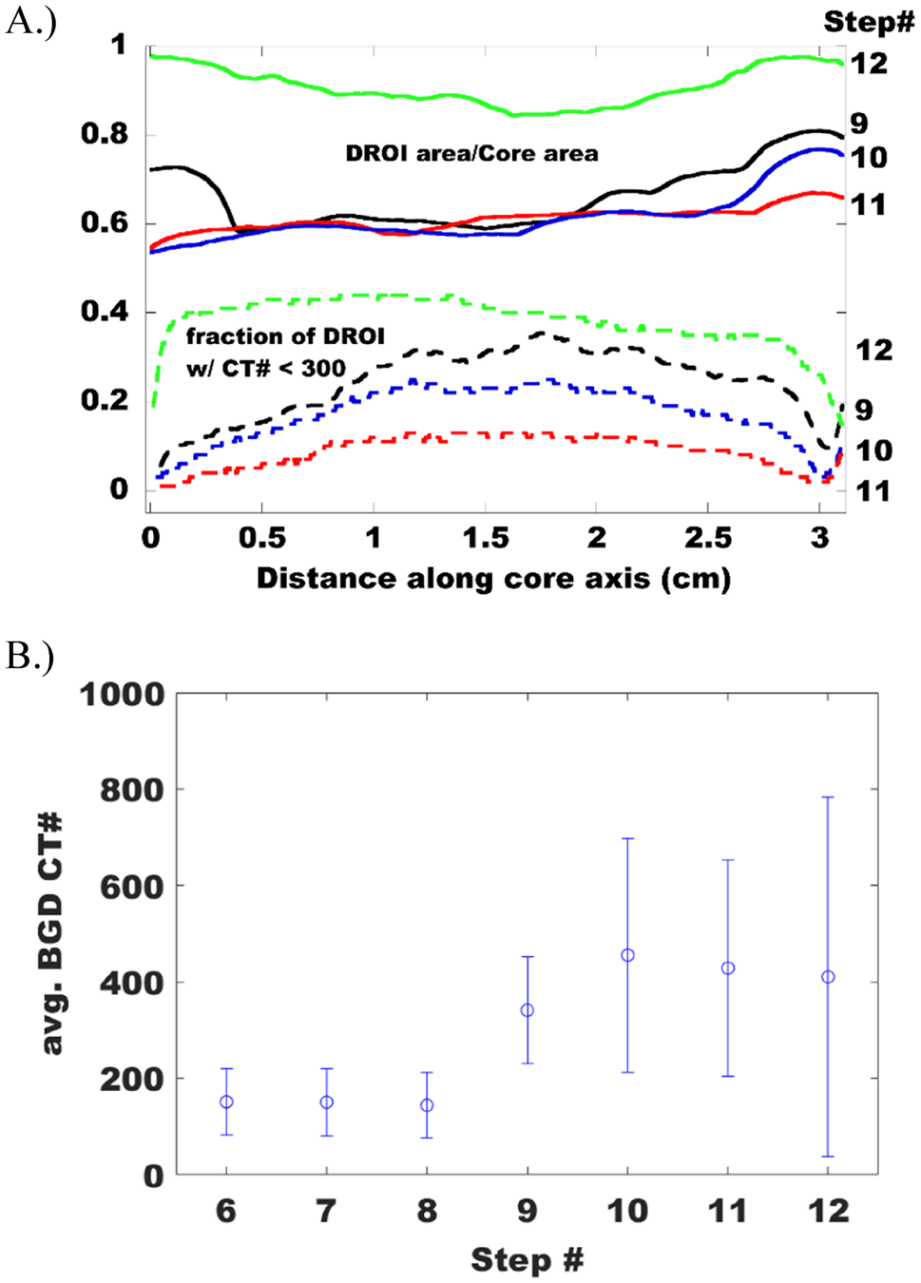
(**A**) *Solid lines*: Ratio between the diffusive region of interest (DROI) and the core cross-sectional area along the length of the core. *Dashed lines*: fraction of the DROI occupied by CT number less than 300 along the length of the core. The product of the two indicates the fraction of the DROI with CT number less than 300 normalized by the core area. (**B**) Average background (BGD) CT number and the breadth of observed values as indicated by the error bars which correspond to 2.5 standard deviations.

While these qualitative observations are instructive, we employed a physics informed machine learning analysis of the images to quantify the proportion of the core accessible through the delaminations, then interrogated that sub-volume to elucidate the mechanisms active during fluid production. First, conventional spectral analysis techniques were used to determine the average and maximum length scale accessible via a delamination as 0.2–0.3 cm, and 0.75 cm, respectively (Fig. 2D). In addition, we identified every pixel associated with a delamination using a recursive user informed classification scheme that employed a deep learning convolutional neural network. The spatial distribution of the delaminations, and the maximum peak width were used to identify every voxel in the diffusive region of interest (DROI) using a 3D morphological filter. A schematic representation of this process is shown in Fig. 2A–D. With all voxels in the DROI identified we analyzed the spatial distribution of Xe throughout the core. The outcome of that process is shown in Figs. 5 and 6 for the three steps that constitute fluid production (step 9–11), and the final step which considers the effect of reducing the confining stress (step 12).

For all stress states the concentration profiles adjacent to the delaminations (e.g. in the DROI) are consistent along the length of the core. This suggests that mass transport through the delaminations was afforded over the entire ~ 3 cm core length. Comparing the Xe profiles for steps 9 and 10 (Fig. 5), when the effective pressure increased by ~ 14 MPa, the most notable change is the concentration profile steepens in the immediate vicinity of the delaminations. There is little change in the concentration gradient beyond 0.1 cm from the delaminations. The relatively flat concentration profiles indicate the pressure equilibrates along the core axis. Therefore, it is impossible to distinguish if the gas accumulating at the delamination migrated from the matrix outside of the DROI (e.g. compression of the matrix), or was simply forced into the DROI due to compression of the delamination. As *P*_eff_ was again increased by ~ 14 MPa (e.g. step 10–11) the system exhibited a somewhat similar response. Xe concentrated immediately adjacent to the delamination, although the concentration perturbation also extended throughout the DROI. In this case, the evolution of Xe concentration outside of 0.2 cm was more subtle, but still readily apparent. Finally, *P*_eff_ was reduced by dropping *P*_conf_ by 14 MPa, accelerating drainage of the DROI. Outside of 0.2 cm, most of the Xe drained from the bulk matrix, and the concentration profile flattened in the immediate vicinity of the delamination. In this case, gas migrated out of the porosity through the delaminations. It is important to note that the data in Fig. 5 indicate that mass transport through the rock matrix occurs in a direction that is perpendicular to the primary external pressure drop along the length of the core. This accentuates the role that the delaminations play in governing access to the porosity in these rocks.

To complement the information in Fig. 5 we also analyze the statistics of Xe concentration in the background rock matrix, or the volume of the core not occupied by the DROI. Under conditions indicative of fluid production (Fig. 1A, steps 9–11), we find that the DROI occupies 60 – 70% of the core volume (Fig. 6A—solid curves). Moreover, this data indicates that the DROI volume evolves near the faces of the core, with most of the evolution occurring in the final 0.5 cm of the core adjacent to the downstream side of the sample. The apparent DROI volume decreases although the number of delaminations identified in our spectral analysis increased by 15% (Fig. 2D, steps 9–10). This apparent contradiction is explained by the observation that most of the new delaminations reside in DROI corresponding to step 9. This observation is also consistent with the mottled appearance of the difference images that suggests non-uniform access to the rock matrix. In the final steps corresponding to hydraulic fracturing (Fig. 1A, steps 6–8, *P*_eff_ = 3.45 MPa), a small volume of Xe displaces the N_2_ in the pore space and we observe a consistent, and low background CT intensity of 150 (Fig. 6B). The leak between step 8–9 causes *P*_eff_ to increase then restabilize at the lowest value, which causes a slight increase in the average background intensity and overall range. Sequentially increasing *P*_eff_ by ~ 14 MPa between steps 9–10, then 10–11 causes an increase in both the mean and range of the background CT intensities. At the same time, the overall DROI volume decreases over roughly one-third of the core length (Fig. 6A, between 2 and 3 cm). Taken altogether, mass transport is spatially non-uniform. Some delaminations are drained (Fig. 6A, between 2–3 cm), the average concentration of Xe in the DROI increases (Figs. 5, 6—dashed curves), and the background CT intensity range increased due to both a slight increase and decrease in the Xe concentration (Fig. 6B). The primary response of the system to increasing *P*_eff_ was to concentrate fluid into the region adjacent to the delaminations. Based on these observations, it can be concluded that the dominant physical mechanisms controlling fluid movement are closure of the delaminations and compression of the disparate matrix. The only strong evidence for fluid production occurs at step 11, which is characterized by the highest *P*_eff_ = 31 MPa. Under these conditions the mean background Xe concentration decreases, accompanied by a decrease in both the maximum and minimum background concentration, and a reduction in the DROI volume (Fig. 6). Fluid is most efficiently produced throughout the core only after *P*_eff_ is reduced (step 12), which results in more uniform access to the matrix (Fig. 6A—solid green curve), and ultimately drained 30–35% of the DROI throughout the rock (Fig. 6—dashed green curve). However, in this case there is still porosity that is not easily drained as indicated by the increase in the maximum Xe concentration observed in the background (Fig. 6B—step 12). It is worth noting that the data corresponding to step 12 in Figs. 1B and 6B indicate that when pathways for fluid production exist, all Xe is removed from the porosity connected to these transport pathways. Therefore, it is unlikely that gas adsorption is responsible for the residual Xe saturation under the thermodynamic conditions imposed throughout our experiments.

**Figure 7:**
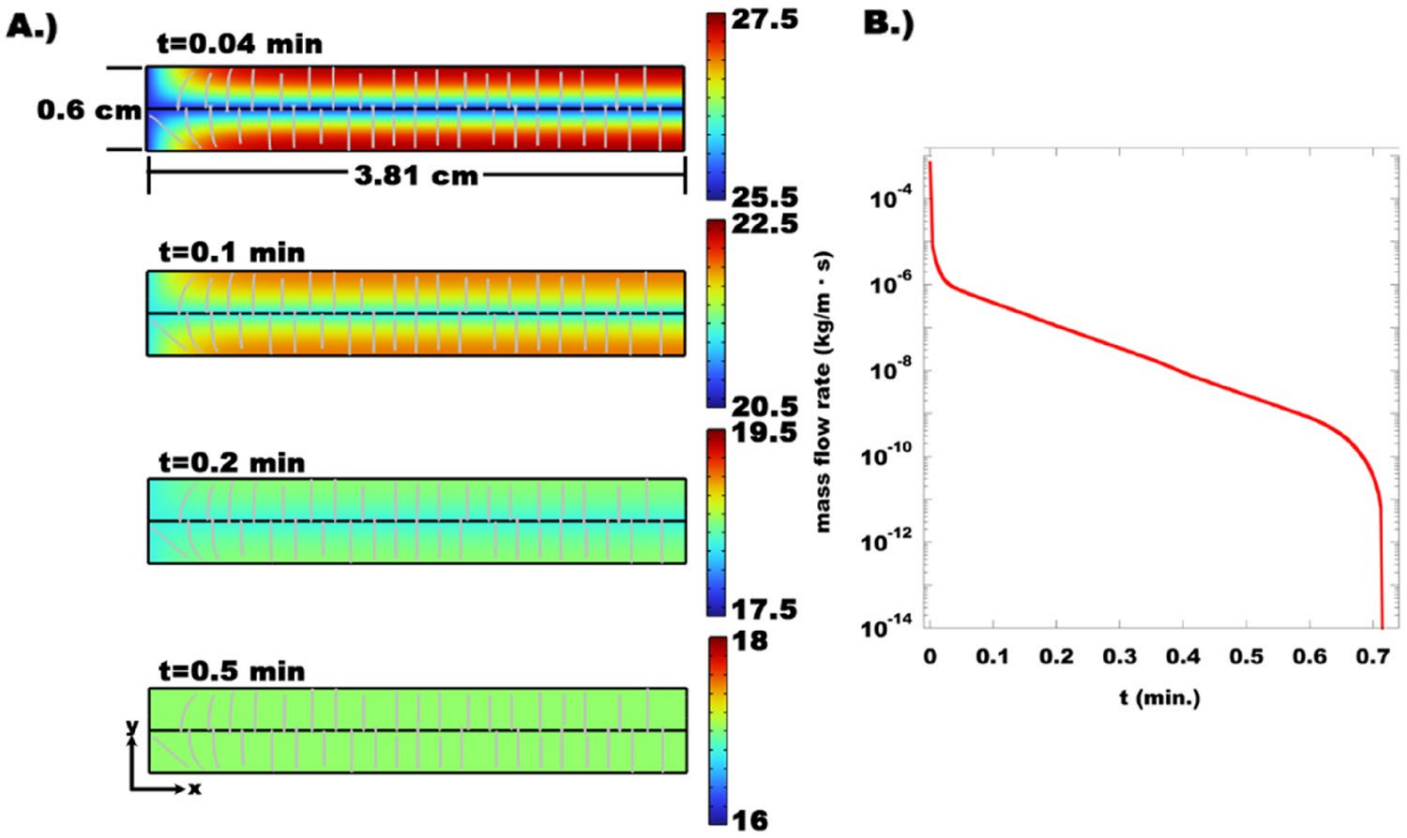
Results of 2D Numerical model to determine the (**A**) Pore pressure through the sample cross-section after a sudden 14 MPa pressure drop at x = 0. The data show the pressure gradient in the vicinity of the delamination relaxes in less than a minute. Fluid flow, as indicated by the gray streamlines is along the sample radius, perpendicular to the global pressure drop along the sample axis. The size of the arrow is proportional to the local flow rate, and the orientation indicates flow direction. At early times fluid flows into, and out of, the crack (black line, centered along the sample length). The only flow trajectories with an x-component are those that lie within the first cm of the face where the pressure drop is imposed. (**B**) mass flux through the face at x = 0. Consistent with the evolution in the pressure gradient throughout the sample, the mass flux drops substantially throughout the first minute, ceasing around 0.7 min.

Finally, we perform 2D numerical simulations using Comsol to model the flow in the vicinity of a delamination. The model consists of a delamination crack with 0.3 cm of porous medium on either side. The relevant thickness of the porous medium was determined from the delamination density in the elemental maps (Fig. 3) as discussed in^9^. The geometry (Fig. S-6), constitutive equations for the poromechanical response, parameter estimation, and associated boundary conditions necessary to mimic the experiment are discussed at length in the supplementary information. Estimates of the material properties, and delamination characteristics, including the pressure dependent delamination aperture for an analogous argillaceous mudstone, were obtained from^9^. Consistent with the experimental observations the numerical results (Fig. 7) indicate that 1 cm beyond the face where *P*_Pore_ is lowered the fluid flows along the cylinder radius towards the delamination. Within the first cm, the flow trajectory evolves from having a component oriented along the sample axis, to along the sample radius only. The numerical results also indicate that fluid is both pressed into, and out of the delamination. These observations are consistent with the experimental observations that fluid concentrates in the vicinity of the delaminations, and that production occurs within a cm of the face where *P*_Pore_ is lowered. Due to the short length scale this process rapidly approaches equilibrium after a change in pressure (Fig. 7B). In fact, the numerical results indicate that no fluid is produced, and the pore pressure in the vicinity of a delamination equilibrates after 0.7 min. This is significantly shorter than the shortest equilibration time imposed before initiating the CT scans during steps 10–12.

In summary, access to the porosity is facilitated by microscale delaminations that lie in bedding planes that are lean in silica and rich in organic carbon. These features are readily accessible at effective pressures consistent with that experienced during hydraulic fracturing. In addition, when the porosity is initially occupied by a liquid at *P*_Pore_, fluid mixing may be accelerated by slowly cycling *P*_Pore_. Upon exposure to a stress state consistent with that meant to afford fluid production (e.g. lower *P*_Pore_, and increase *P*_eff_) transport in the system quickly arrests with the majority of the pore fluid trapped in a region that is within 0.2 cm of the delamination. The maximum fluid production is on the order of 5–10% of the DROI volume, and this occurs from the regions of the core located closest to the free surface where the boundary condition is imposed. The fluid content in the central portion of the core is not affected by a reduction in *P*_Pore_. This behavior is due to the concomitant closure of the delaminations and compression of the background rock matrix. More efficient fluid production is observed when *P*_conf_ is subsequently reduced, permitting a large proportion of the porosity to empty through dilated delaminations. This work is the first to demonstrate that cm-scale mass transport in oil bearing mudstones is governed by migration through delaminations, which is clearly effective pressure dependent. Therefore, fluid production may be enhanced by techniques to access these indigenous weak planes and prevent subsequent closure when the effective pressure increases. Moreover, care must be taken when measuring pressure dependent properties, like permeability, at the core scale, and utilizing this information in simulations at the reservoir scale. In short, the upscaling of this behavior remains an open question; Ultimately more work is necessary to provide guidance on the volume of rock accessible through delaminations at larger length scales in order to make more accurate field scale predictions. Finally, even without optimization, a significant proportion of the rock porosity is accessible under conditions that mimic fluid injection. Coupled with the observation that fluid is trapped when *P*_eff_ is subsequently increased, this indicates that these reservoirs have potential to securely sequester CO_2_.

### Supplementary Information

Supplementary Figures.

## Data Availability

The raw data are not publicly available due to ExxonMobil policy. The raw data that support the findings of this study are available upon reasonable request from the corresponding author JJV, subject to approval from ExxonMobil.
