# Supplementary material for: Fracture-matrix fluid exchange in oil-bearing unconventional mudstones
Source: Sci Rep. 2023 Dec 7;13:21708. doi: 10.1038/s41598-023-48688-z (PMC10709332; doi:10.1038/s41598-023-48688-z)
Supplement: Supplementary file 1 — Supplementary Figures. [file 41598_2023_48688_MOESM1_ESM.pdf]

Supplementary Information

X-ray Diffraction (XRD)

X-ray diffraction (XRD) was performed on reservoir materials using a PANalytical X'Pert Pro diffractometer. An aliquot of each sample was powdered with a shatter box and spiked with corundum to ensure accuracy when determining mineral phase mass fractions. The measured mass fractions were inferred from the x-ray spectra using the Rietveld profile fitting method. The composition of the samples utilized in this study are shown in **Fig. S- 1**. Also shown is a comparison to data from a similar study, and the expected compositional envelope for rocks from the Permian basin.

Mercury Intrusion Capillary Porosimetry (MICP)

Mercury intrusion was performed with a micromeritics Autopore IV 9500. Prior to the measurement, the rock was granulated and sieved to yield a sufficient quantity of particles with a diameter in the range 0.1 – 0.3 cm. The mercury intrusion volume was logged at 205 pressures in the range 0.003 – 413.6 MPa. The Washburn equation, with known values of the contact angle and surface tension of mercury/air [30], was used to relate pressure to the pore volume accessible through pores that range in size from 3.60 to 0.003 microns, respectively. Results for the rocks utilized in this study are shown in **Fig. S- 5** along with data collected on a conventional Berea sandstone. The results indicate that unconventional reservoir materials are characterized by a significant reduction in porosity, and pore size.

Electron probe microanalysis (EPMA) and Scanning Electron Microscopy (SEM)

Sample preparation

Prior to measuring the elemental composition or acquiring images with the SEM a ~3 mm thick disc was removed from each core (see **Fig. 3** – main text). After affixing the disc to a circular steel sample holder, one face of the disc was polished with a Buehler EcoMet 250 under 4-lbs of compressive force. Initially the samples were coarse polished with diamond embedded abrasive sheets in n-heptane. The abrasive grit was sequentially increased from 180 (78 μm) to 1100 (8 μm). The polished surfaces were finished with Buehler TriDent polishing cloth using 6, 3, then 1 μm diamond sprays (in alcohol). Finally to prevent charging from the electron beam, the sample was carbon coated under vacuum. We estimate that the carbon coating is about ~20 nm thick.

EPMA

The measurement consists of rastering a 15 keV electron beam over the polished surface while detecting the x-rays emitted from the surface minerals with dual energy dispersive x-ray spectroscopy (EDS) detectors. The spot size of the beam is on the order of 1 μm, but the pixel size is estimated to be 4.97 μm for each image. This reflects the fact that the electrons scatter in the near surface volume and interact with atoms not immediately under the illuminated spot. The final measurement consists of an image that is up to 1 cm wide by 3.5 cm tall. To interrogate such a large area, we stitched together measurements on smaller areas (256 × 190 pixels or ~0.13 x 0.094 cm). The dwell time on each pixel is 50 μs, and each area is scanned 125 times and averaged, so each small area is scanned in about 5 minutes, and the total area takes nearly a day to scan. After averaging, the x-ray spectra are converted to elemental mass fraction with a matrix corrected ZAF approach [31], using specific elemental standards that are consistent with the predominant elements in our samples. The mineral standards we used were, Al_2O_3, $CaCO_3$, NaCl, FeS_2, K-feldspar, MgO, SiO_2, FeP, and TiO_2. Assuming earth materials are predominantly composed of these minerals we determine the elemental composition consisting of Al, Ca, C, Cl,

Fe, K, Mg, Na, O, S, Si, and Ti. Of these we use Al, C, Ca, Fe, Si, as proxies for the major phases expected in mudstones, those being, clay, organic or inorganic carbon, pyrite, and silt/sand, respectively.

SEM

An FEI Quanta 600 environmental scanning electron microscope (SEM) equipped with (EDS) was used to characterize microstructure and elemental composition, respectively. The X-ray spectra were gathered with a backscattered detector then analyzed to determine the elements irradiated by the electron beam. In addition, a secondary electron detector was used to observe topography and morphological features. Large structural features (e.g. natural fractures, organic/mineral distributions) were observed using low magnification (100x) montage imaging techniques. Low to high magnification (50x to 20,000x) was utilized in several locations to characterize the rock composition.

Coupled poroelastic rock matrix/microcrack response

The coupled poroelastic response of a porous matrix adjacent to a more compliant crack is a challenging analytical pursuit. Alternatively, the response may be estimated through numerical solution of the governing equations subject to appropriate initial and boundary conditions (e.g. relevant to our experiment). To this end we employ Comsol poroelastic module, which in turn uses solid mechanics to account for the elastic response of the porous matrix, and Darcy's law to describe fluid flow. The simple 2D geometrical model in question consists of a compliant delamination crack surrounded by a porous matrix as shown in **Fig. S- 6**. The length of the model is consistent with that of the samples used in this study, and the thickness of the porous matrix was determined from the delamination density as discussed in reference 9. The delamination is added to the geometry using the 2D fracture network add-in from the subsurface flow module. The delamination is designated as a highly conductive fracture.

The governing equation in the solid mechanics model is that of mechanical equilibrium under plain strain conditions

$$\nabla \cdot S = 0 \qquad \text{(S-1)}$$

where S is the second Piola-Kirchhoff stress. We use a linear elastic constitutive model defined by the Young's modulus, E=37.5 GPa [9], Poisson's ratio, ν=0.25 and material density, ρ_S=2410 kg/m^3 (**Table 1**). Consistent with the experimental conditions, we impose a constant distributed force boundary condition on all four boundaries of the model, where the force per unit area is equivalent to the confining pressure:

$$F / A = P_{conf} = 34.5 \text{ MPa} \qquad \text{(S-2)}$$

It is necessary to distinguish between force and pressure at the boundary to avoid the implication that the pressure at the boundary is in communication with the pore fluid. In the experiment, this boundary condition is imposed through the Buna-N sleeve, and the end caps for fluid distribution. The initial material displacement was set to zero throughout the volume being modeled. The concomitant fluid flow accounts for the volumetric strain in the porous solid, ε_P, and the flow generated by the imposed pressure gradient, ∇P,

$$\begin{aligned} &\frac{\partial}{\partial t}\left(\varepsilon_P \rho_L\right) + \nabla \cdot \left(\rho J\right) = 0 \\ &J = \frac{\kappa}{\eta} \nabla P \end{aligned} \qquad \text{(S-3)}$$

where the second of Eq. S-4 is Darcy's law, J is the flow velocity and κ is the porous matrix permeability. The pressure dependent permeability of the porous matrix was estimated from the Carmen-Kozeny equation given the pore size and porosity shown in **Table 1**, $\kappa \approx \phi r_P^2/5 \approx 20\times10^{-20}$ m^2 (e.g. at the low P_{eff} limit). We account for a 40% reduction in matrix permeability over the modeled P_{eff}. The fluid density, ρ_L, and viscosity, η, are set to the pressure dependent values for Xe as reported by the National Institute for Standards and Technology (NIST) chemistry webbook (https://webbook.nist.gov/chemistry/fluid/). The permeability of the crack is given by the slit model:

$$\kappa_C = \frac{b^2}{12} \qquad \text{(S-4)}$$

where b is the crack aperture. In reference 9 we show the crack compliance is much greater than the surrounding matrix. The associated effect on the crack permeability is accounted for in Equation (S-4) through an expression for the pressure dependence of the crack aperture, which was derived in reference 9:

$$b = -12.4\times10^{-6}\ln\left(\frac{P_{eff}}{37.5\times10^{6}}\right) \qquad \text{(S-5)}$$

The initial pressure throughout the volume being modeled was set to P_{pore}=31 MPa. There was a temporal pressure change imposed at x=0 to represent a step change in the pore pressure:

$$P_{pore}(x=0) = 31\times10^{6} - 14\times10^{6}(1-e^{-t/5}) \qquad \text{(S-6)}$$

A mass flux boundary condition, **-n•ρu=0** (no flow)**,** was imposed on all other boundaries.

All that remains is to account for storage of the fluid in the porous medium and the crack due to concomitant compression of the fluid, and the solid phase. We assume there is no storage in the crack, so the fluid squeezed out of the crack is simply given by the change in the crack porosity due to a reduction in the crack aperture. We set the initial crack porosity to ϕ_C=0.7. In the porous medium the fluid storage is given by the Biot parameter which we set equal to α_B=(1-K_P/K_S)=0.6 based on our observations of the porous medium (1/K_P) and solid constituent (1/K_S) compliances in reference 9.

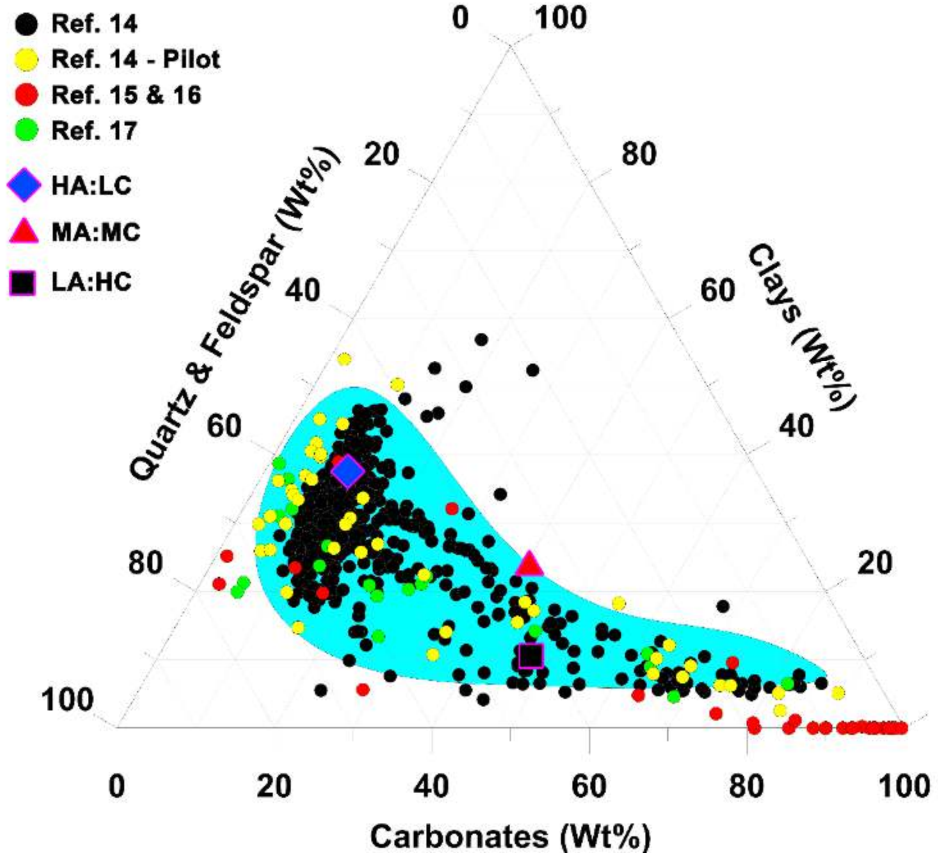

Fig. S- 1 - Ternary diagram showing compositional envelop inferred from the indicated references. The larger non-circular shapes, highlighted in magenta, indicate the sample composition determined by x-ray diffraction (XRD – see supplementary information) for the plugs used in this study.

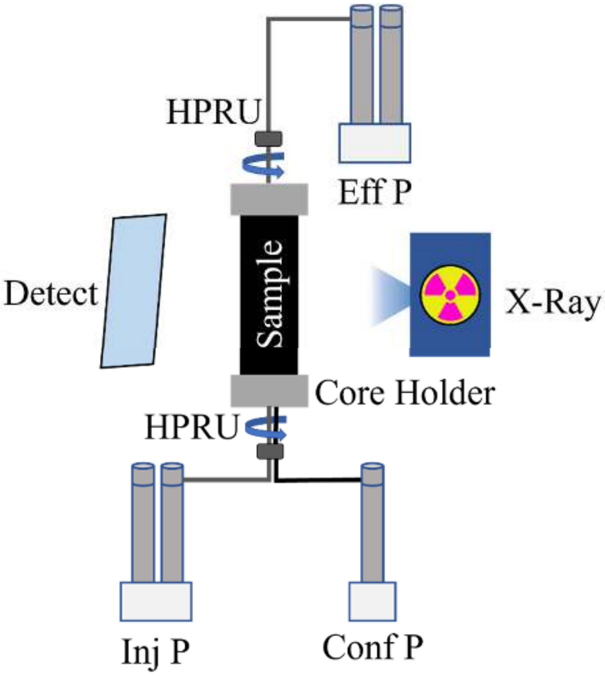

Fig. S- 2 - Schematic of computed tomography scanning and core flow system. Labels: **X-Ray**: Feinfocus FXE Source, 185kV 400 mA. **Conf P**, **Inj P**, and **Eff P**: Teledyne ISCO 100DX Pumps maintaining confining, injection, and effluent pressures. **HPRU**: APSM-130 High pressure rotating unions (Scott Rotary) allowing sample to rotate during CT acquisition. **Detect**: Perkin Elmer 4000 x 4000 pixel flat panel detector. **Core Holder**: TEMCO carbon fiber wrapped X-Ray hassler style core holder. Sample secured in Buna-Nitrile sleeve, inside core holder with floating stainless steel end bells and fluid distribution caps. Confining pressure maintained in annulus between sleeve and core holder, fluid injection controlled from Inj P, and system pressure/effluent captured with the Eff P.

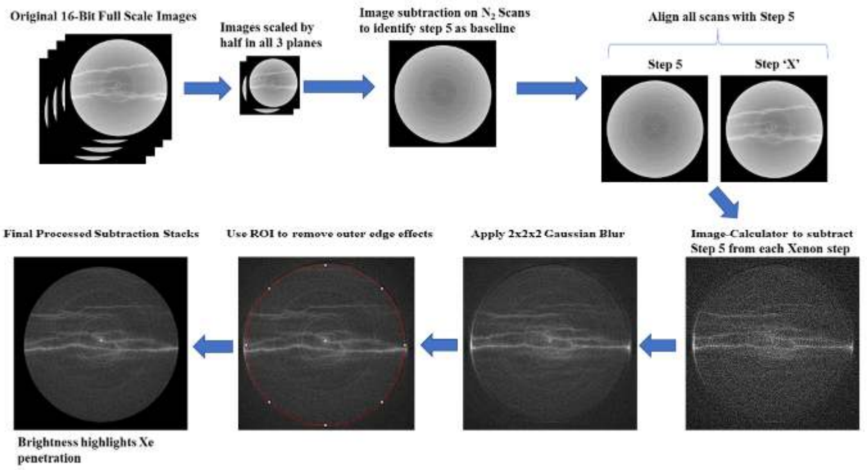

Fig. S- 3 – X-ray CT Image processing to highlight voxels enriched with the contrast agent, Xenon. As indicated in the text, substituting Xe (Z=54) for N_2 (Z=7) highlights the accessible rock volume due to the increase in x-ray absorption.

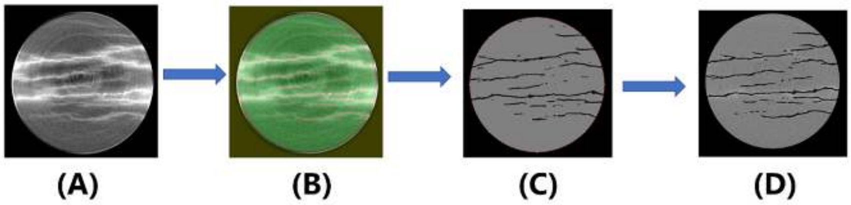

Fig. S- 4 - Deep learning on an individual CT slice to classify voxels associated with the rubber sleeve/air (black pixels outside of the circular cross section), matrix (gray pixels), and fractures (black pixels inside the cylindrical cross section). (A) 16-bit greyscale image from CT scan, (B) Classification from *ilastik* (note opacity shading between fracture and matrix zones), (C) segmented fractures and matrix, and (D) isolated fractures overlaying 16-bit greyscale image.

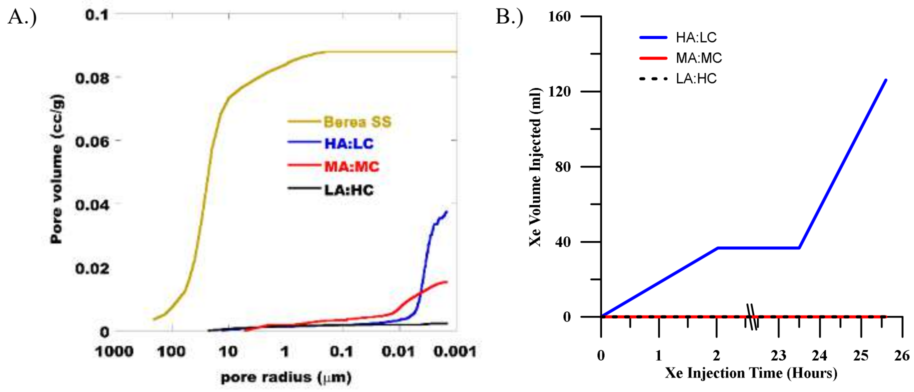

Fig. S- 5 - A.) Cumulative mercury intrusion volume for a Berea sandstone (SS), and the tight rocks considered in this work. B.) Volume of Xe transmitted across the rock core for all three samples corresponding to step 6 – 9 (**Fig. 1**).

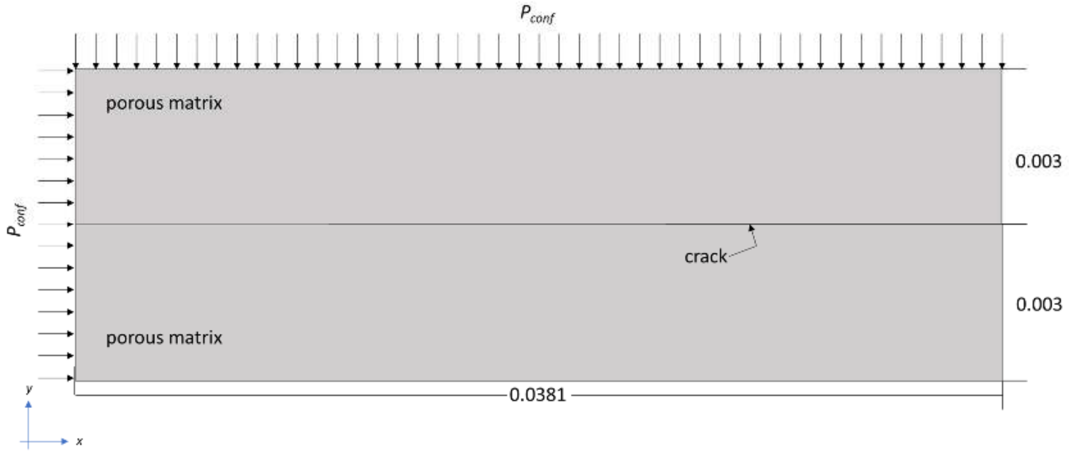

Fig. S- 6 – Simple geometrical model utilized in COMSOL calculation with indicated spatial dimensions. Indicated boundary pressure is applied on all exterior sides of the model.
